# Supplementary figures and images for: The MAPK substrate MASS proteins regulate stomatal development in Arabidopsis
Source: PLoS Genet. 2020 Apr 2;16(4):e1008706. doi: 10.1371/journal.pgen.1008706 (PMC7156110; doi:10.1371/journal.pgen.1008706)

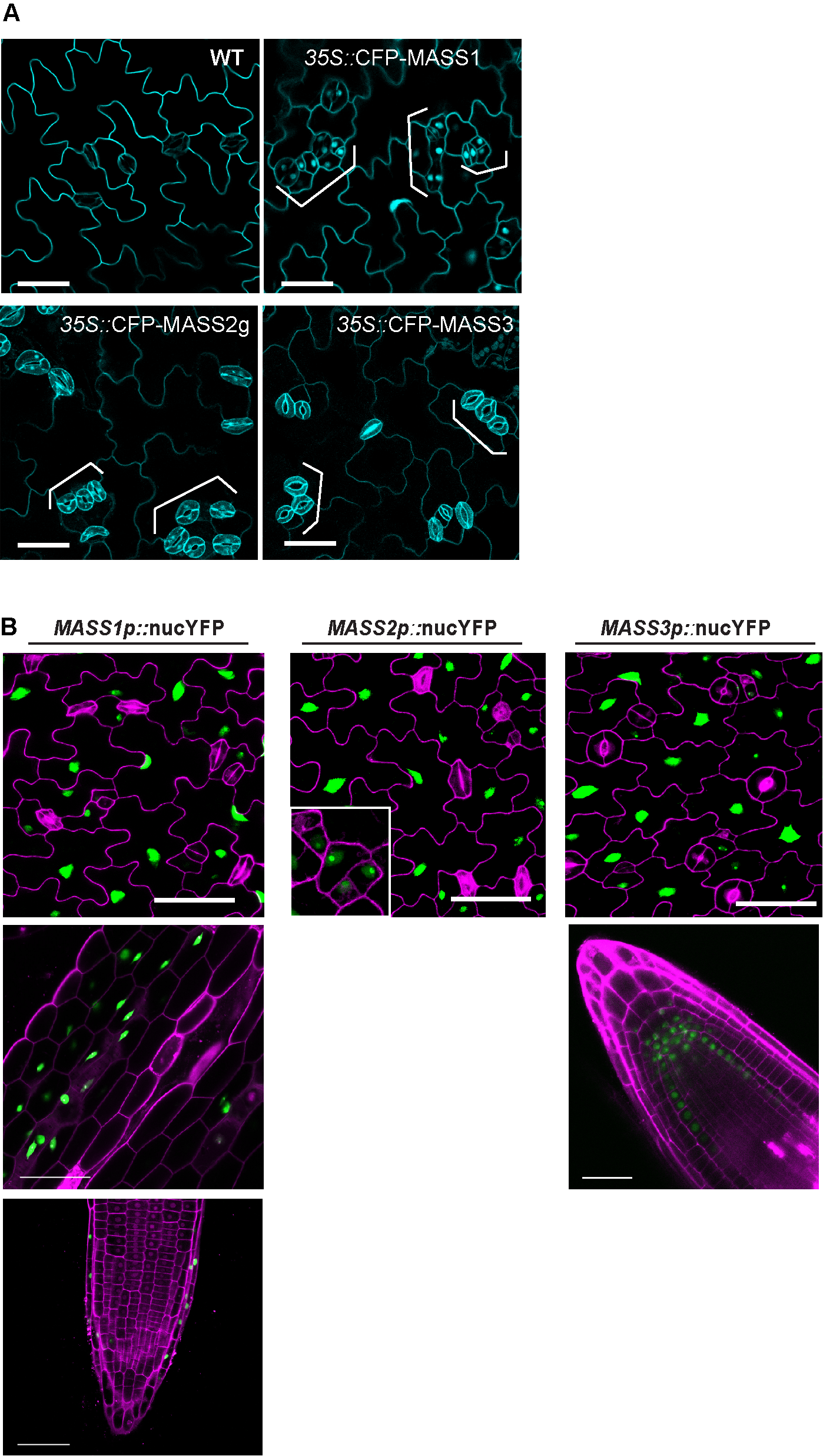

Supplement: S1 Fig — (A) Stomatal phenotype of MASS overexpression lines. Confocal images of 7-dpg adaxial side of the cotyledon epidermis in WT (Col) and CFP-MASS1, CFP-MASS2g, and CFP-MASS3 seedlings, all driven by 35S promoter. Brackets indicate stomatal clusters. Scale bars represent 50 μm. (B) Confocal images to show transcriptional activities of the MASS1/2/3 promoters displayed by the expression of nuclear YFP (nucYFP, green). Cell outlines were stained with Propidium Iodide (PI). The inset showing more detailed expression pattern. Scale bar represents 50 μm in (A) and 20 μm in (B). (TIF) [file pgen.1008706.s001.tif]

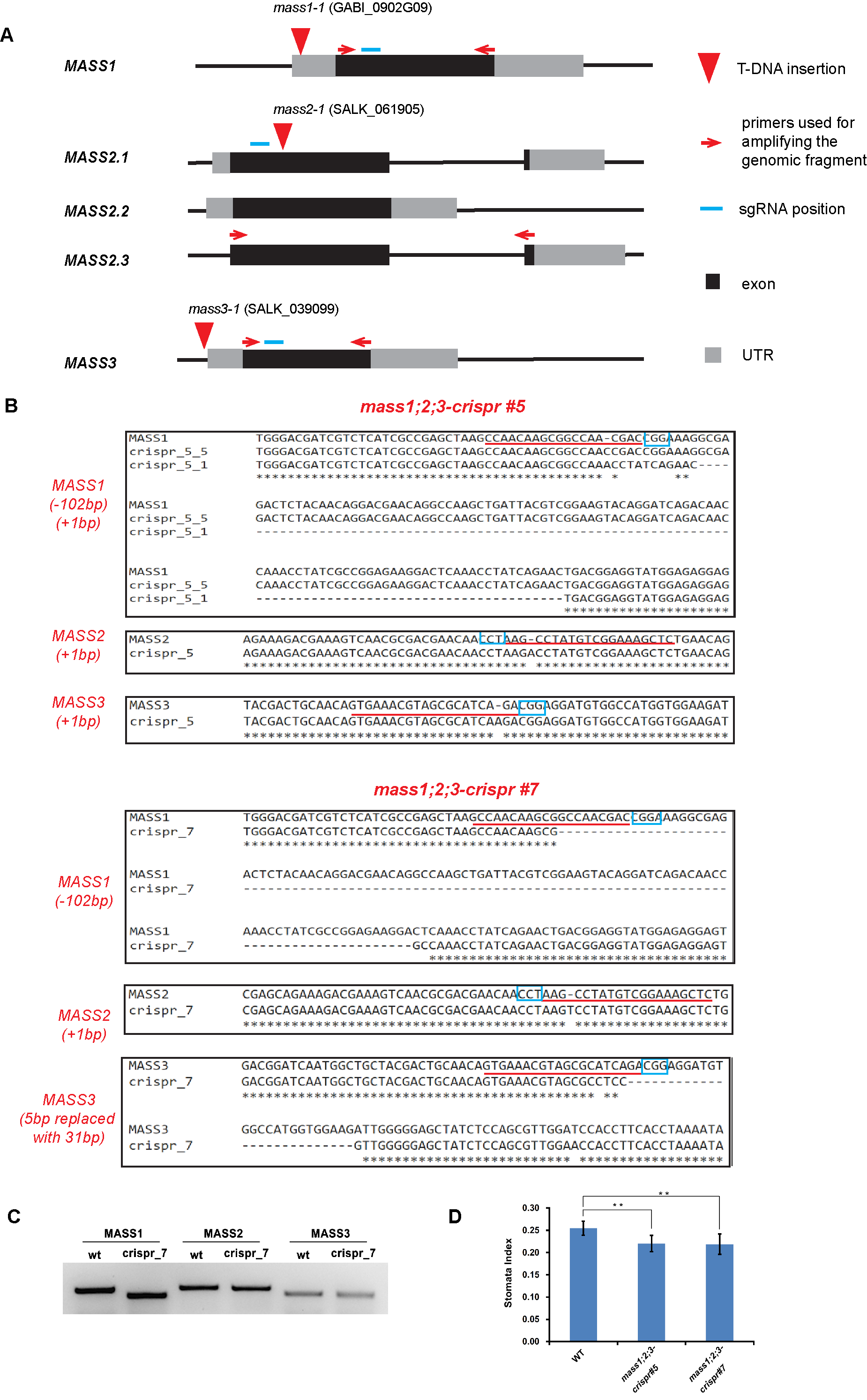

Supplement: S2 Fig — (A) Diagram of the gene structure and splicing variants of the MASS genes. (B) Genotyping results for the crispr mutants. DNA sequence alignments showing the edited DNA sequences of the three MASS gene editing in mass1;2;3-crispr #5 and #7 lines. The PAM sequences were outlined with blue boxes, sgRNAs were underlined with red. (C) Genotyping PCR showing a long deletion in MASS1 in a T3 mass1;2;3-crispr #7 mutant plant. (D) Quantification of SI in 5-dpg adaxial cotyledons of mass1;2;3-crispr #5 and #7 mutant. ** significantly different between the two samples being compared (bars). Student’s t-test, **P < 0.001. (TIF) [file pgen.1008706.s002.tif]

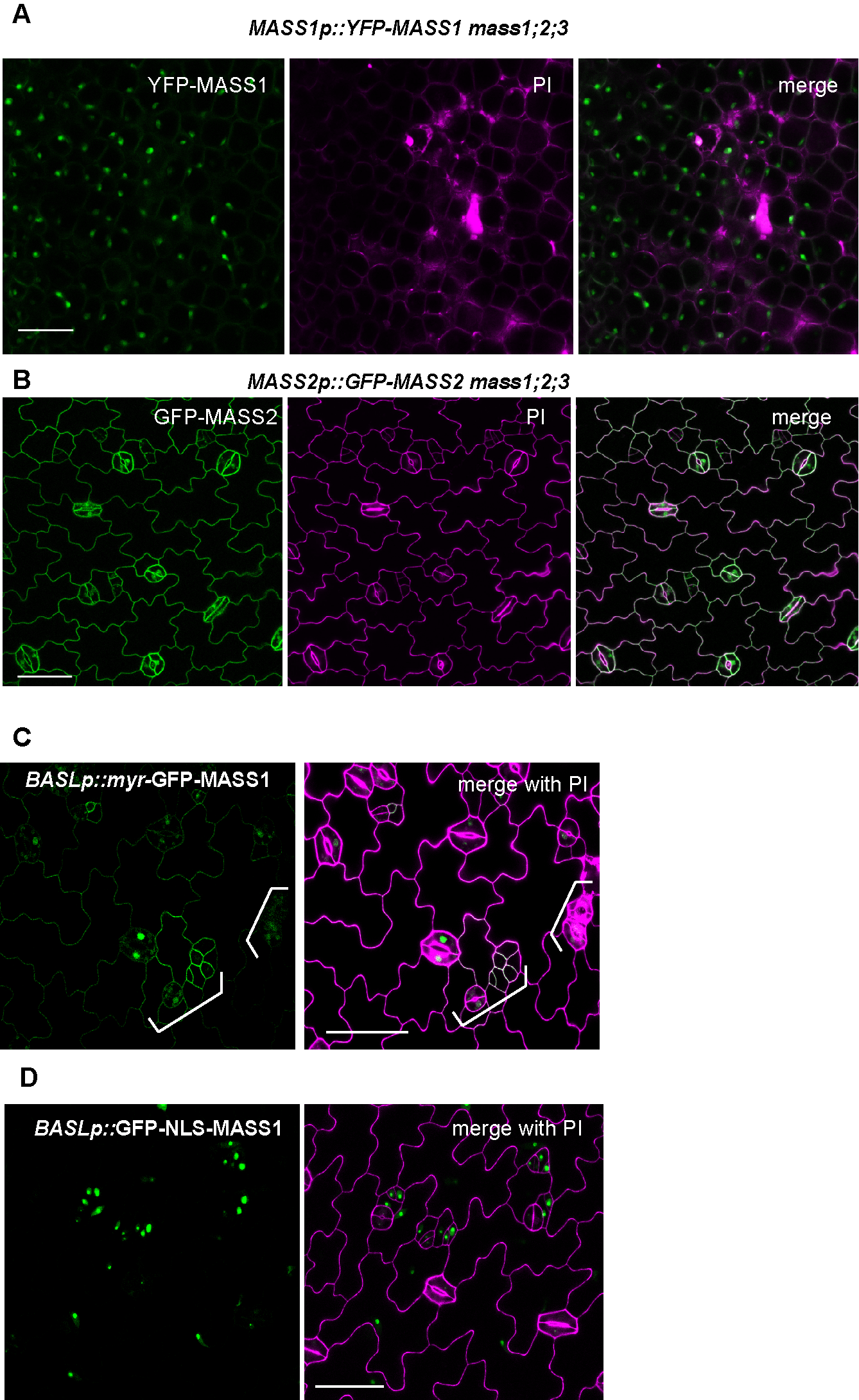

Supplement: S3 Fig — (A) MASS1pro::YFP-MASS1 (B) MASS2pro::GFP-MASS2 in T-DNA triple mutants at 3-dpg. Note, strong signals of YFP-MASS1 in the mesophyll cell layer (A), whilst strong YFP-MASS2 in the epidermis (B). (C, D) Confocal images showing stomatal phenotype in plasma membrane-localized (C) and nuclear-localized (D) GFP-MASS1 seedlings, both driven by the BASL promoter. Green: GFP signals, magenta: cell outlines stained with PI. Left panels show protein localization, right panels show the overlay of green and magenta. White brackets indicate stomatal clusters and abnormal cell divisions. Scale bar represents 50 μm. (TIF) [file pgen.1008706.s003.tif]

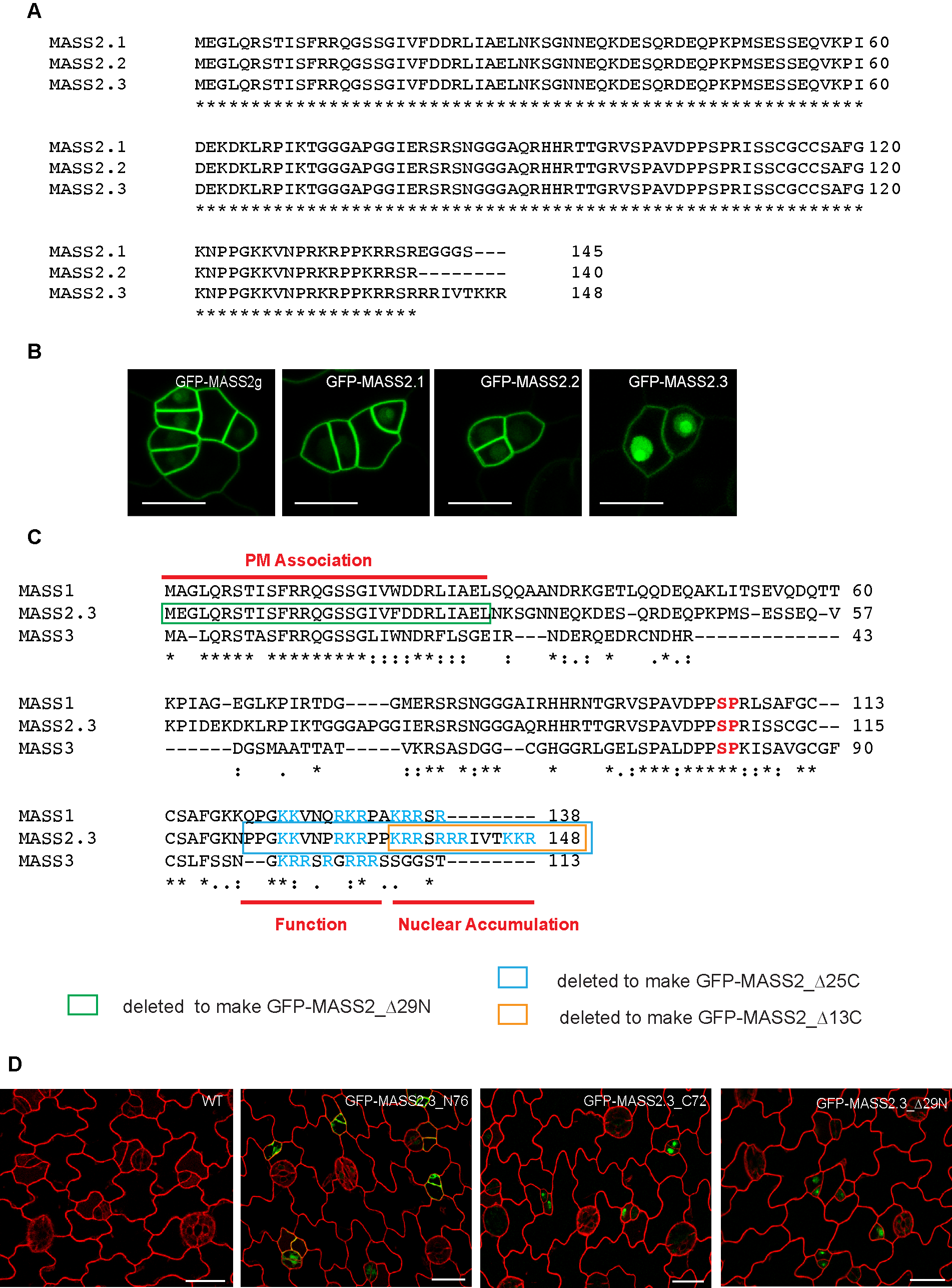

Supplement: S4 Fig — (A) Amino acid alignment of the MASS2 splicing variants. (B) Confocal images showing the detailed localization of GFP-tagged MASS2g, MASS2.1, MASS2.2, and MASS2.3 (green), all driven by the BASL promoter. Scale bar represents 10 μm. (C) Amino acid alignment of MASS1, MASS2.3, and MASS3 and the identified subdomains required for specific functions. The deleted amino acids to make GFP-MASS2.3 truncations were outlined with designated colors. (D) Confocal images of 3-dpg adaxial side of the cotyledon epidermis showing localization and stomatal phenotype of truncated MASS2.3 proteins. Cell outlines were stained with PI. Scale bar represents 20 μm. (TIF) [file pgen.1008706.s004.tif]

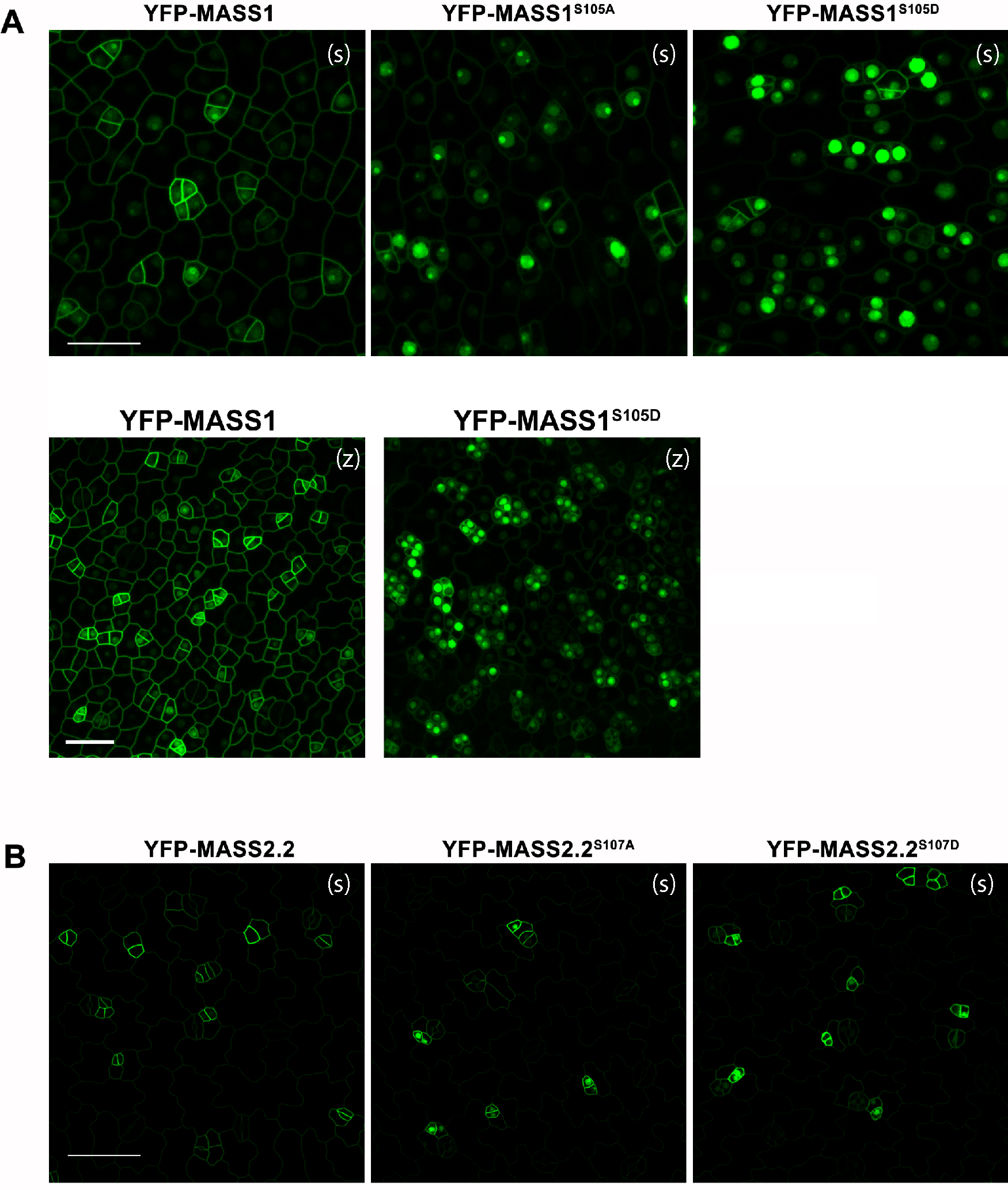

Supplement: S5 Fig — (A) Confocal images of YFP-MASS1 and phospho-variants shown as single optical section (s) vs. the z-projections (z). (B) Confocal images of YFP-MASS2.2 and phospho-variants in single optical section (s). Scale bar represents 50 μm. (TIF) [file pgen.1008706.s005.tif]

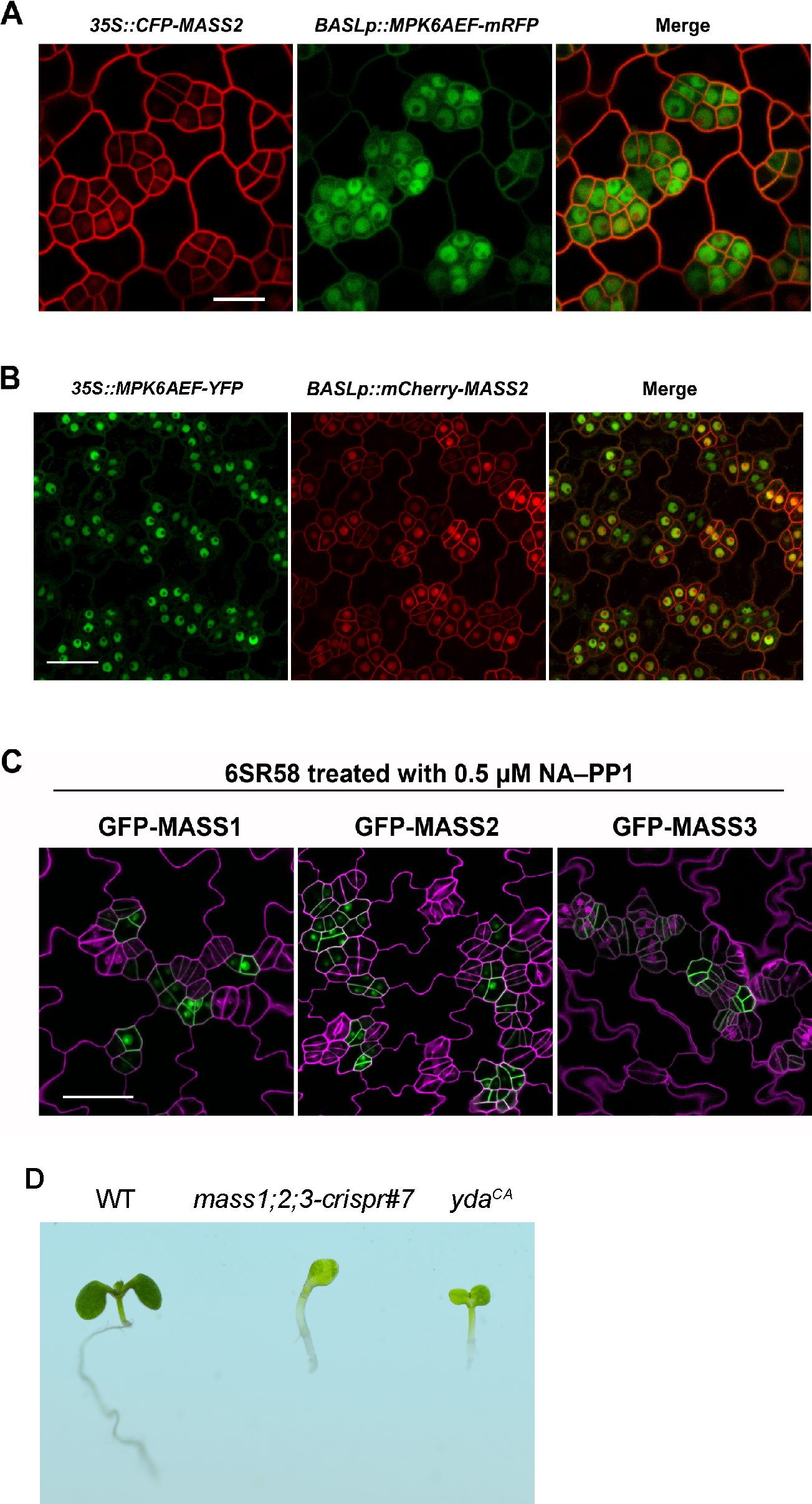

Supplement: S6 Fig — (A) Confocal images to show 35S::CFP-MASS2g (red) co-expression with overexpression of the dominant negative (kinase inactive) MPK6 (BASLp::MPK6AEF-mRFP, green). Scale bar represents 20 μm. (B) Confocal images of BASLp::mCherry-MASS2 (red) co-expressed with 35S::MPK6AEF-YFP (green). Scale bar represents 50 μm. (C) Confocal images of GFP-MASS in chemically inducible MPK6 inhibition in mpk3;6 null background. Scale bar represents 50 μm. (D) 5-day-old seedlings of WT, mass1;2;3 and YDACA. (TIF) [file pgen.1008706.s006.tif]

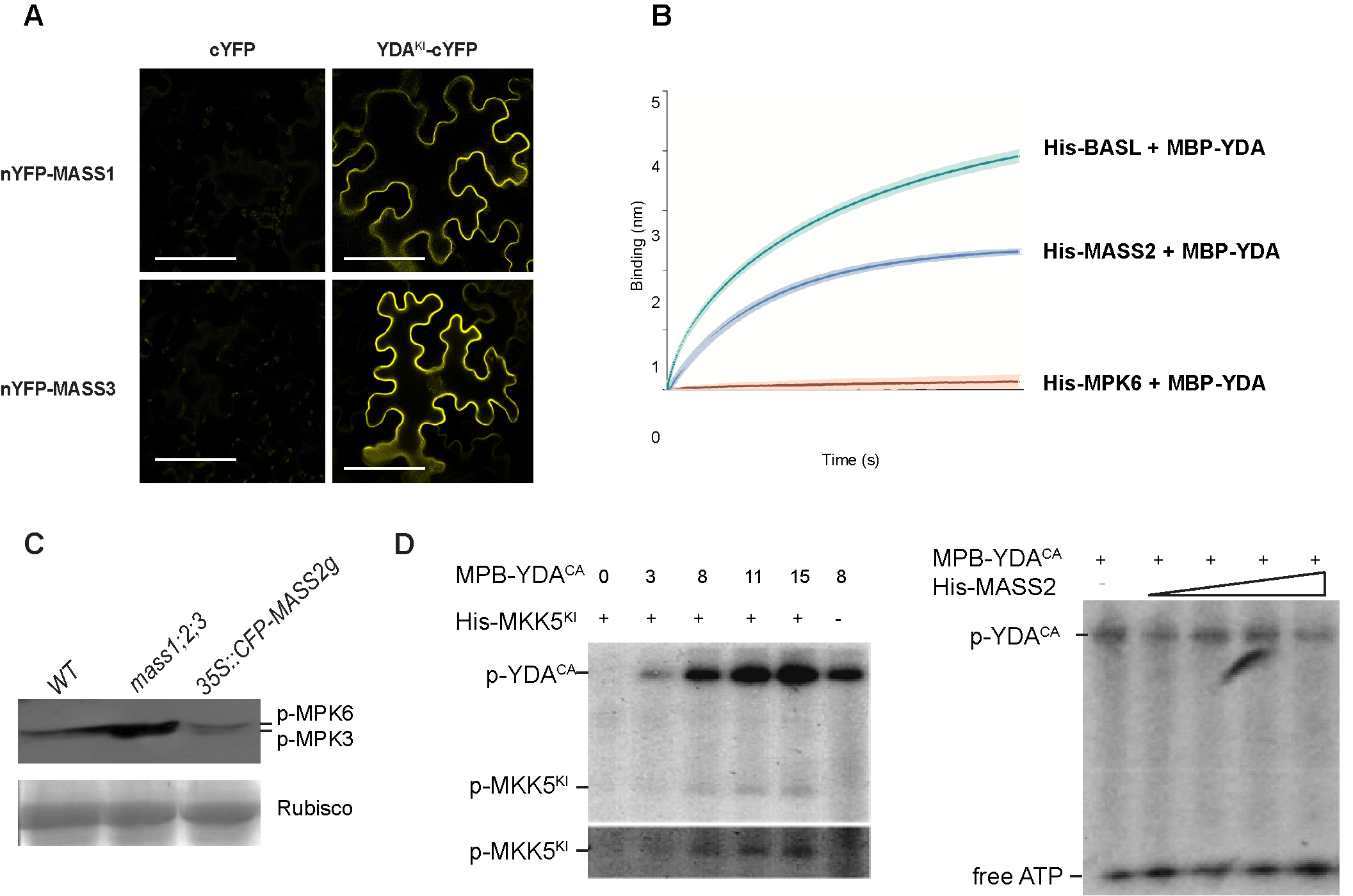

Supplement: S7 Fig — (A) Confocal images to show BiFC interaction tests between YDAKI and MASS1/3 in tobacco leaf epidermis. The expression of half YFP (cYFP) was used as negative control. Scale bar represents 50 μm. (B) BLI tests to show the interaction between YDA and MASS2. The BASL-YDA interaction was used as positive control, while MPK6-YDA as negative control. (C) Western blot to test activated MPK3/6 levels in mass mutants and overexpression plants. (D) In vitro YDACA autophosporylation levels by in vitro kinase assay. Increasing amount of MASS2 was added to test whether it affects YDACA autophosporylation activity. MKK5KI as a positive control, in which elevated levels of YDACA trigger elevated phosphorylation of MKK5. (TIF) [file pgen.1008706.s007.tif]

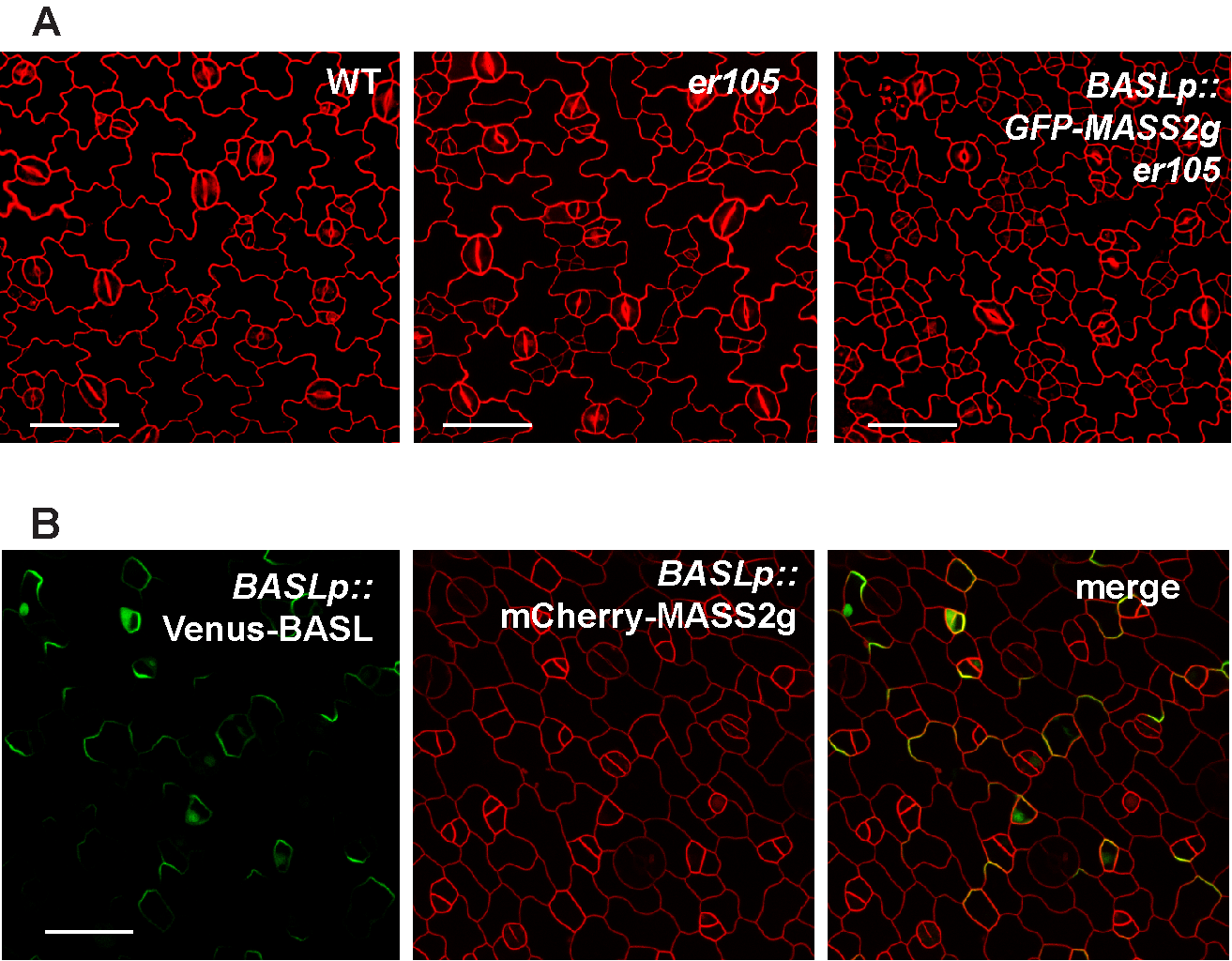

Supplement: S8 Fig — (A) Genetic test between er105 and a GFP-MASS2g overexpression line, driven by BASL promoter. Cell outlines were stained with PI (red). (B) Confocal images showing localization of Venus-BASL (green) and mCherry-MASS2g (red), both driven by the BASL promoter. Scale bar represents 50 μm. (TIF) [file pgen.1008706.s008.tif]

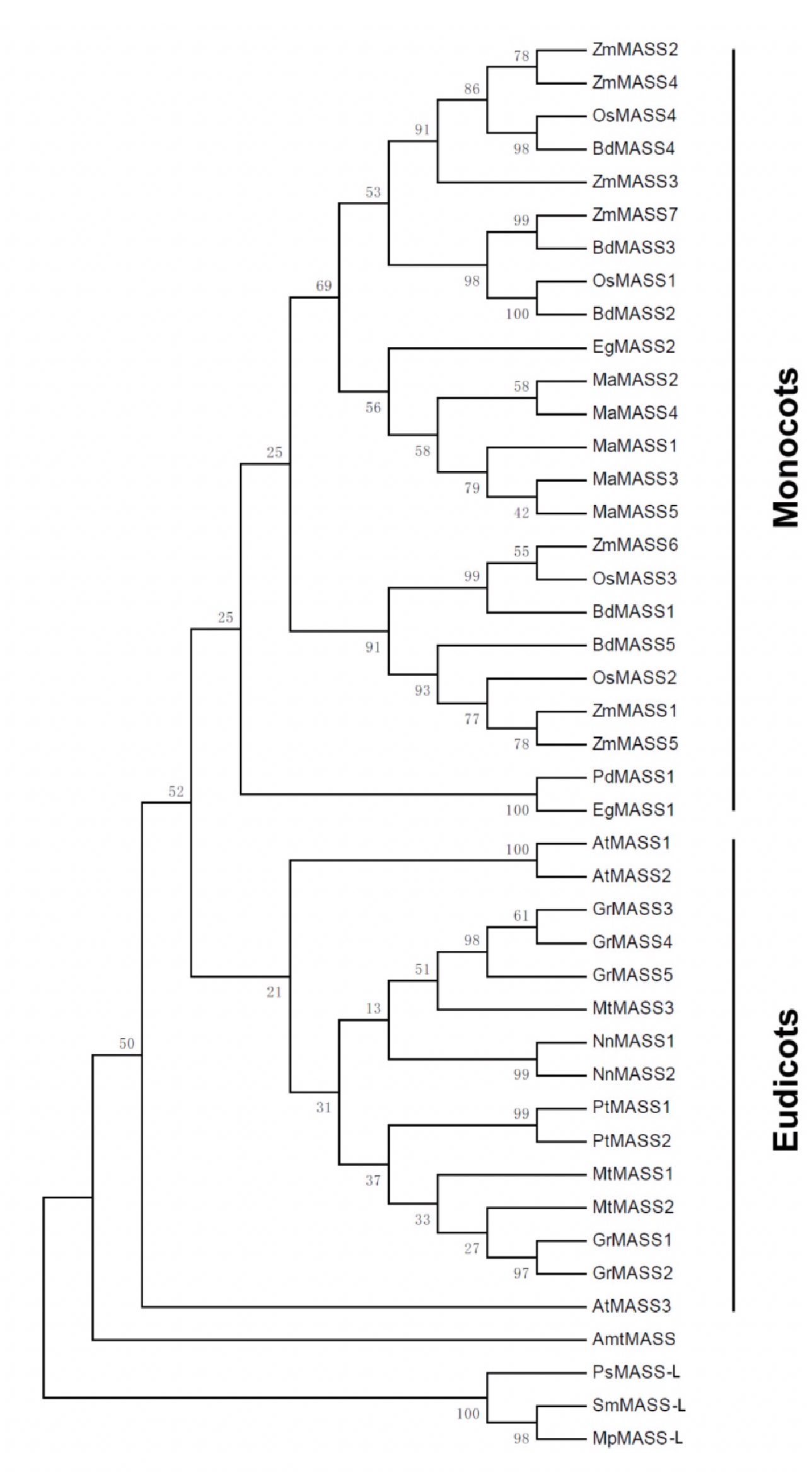

Supplement: S9 Fig — Protein sequences of the three Arabidopsis MASS proteins were compared with those of 40 orthologs retrieved by GenBank blasting representing embryophyta (lycophyte, gymnosperm and angiosperms). Phylogenetic tree was constructed by the program MEGA6 [47] using the neighbor-joining method. The reliability of the phylogenetic tree was evaluated by bootstrapping of 1000 replications. (TIF) [file pgen.1008706.s009.tif]
