# Supplementary material for: The MAPK substrate MASS proteins regulate stomatal development in Arabidopsis
Source: PLoS Genet. 2020 Apr 2;16(4):e1008706. doi: 10.1371/journal.pgen.1008706 (PMC7156110; doi:10.1371/journal.pgen.1008706)
Supplement: S1 Table — (PDF) [file pgen.1008706.s010.pdf]

S1 Table. Primers used in this study

Purpose	Primer Name	Sequence (5'-3')
MASS1 CDS cloning	MASS1N-F-NotI	GCTCCGCGGCCGCCATGGCGGGTCTTCAGAGATC
	MASS1C-R-AscI	AGGCGCGCCCCTATCTGGATCTACGCTTCGCTG
Genomic MASS2 cloning	MASS2-F-NotI	GCTCCGCGGCCGCCATGGAGGGTCTTCAAAGATC
	MASS2-R-AscI	AGGCGCGCCCTTATCTTTTCTTGGTCACGATCCG
MASS3 CDS cloning	MASS3N-F-NotI	GCTCCGCGGCCGCCATGGCGTTGCAGAGATCAAC
	MASS3C-R-AscI	AGGCGCGCCCTTAAGTACTTCCACCGCTTGATC
MASS1 qPCR	qMASS1-F	CGAGCTAAGCCAACAAGCGGC
	80180-R+TAG	CTATCTGGATCTACGCTTCGCTG
MASS2 qPCR	qMASS2-F	ATCTTCCGGTATAGTGTTTGAC
	qMASS2-R	GCAACAACCGCATGACGAAAT
MASS3 qPCR	AT5G20100-F	ATGGCGTTGCAGAGATCAACAGC
	AT5G20100-R	AGTACTTCCACCGCTTGATCTTC
MASS1-CRISPR	80180-CRI-F2	GATTGCCAACAAGCGGCCAACGAC
	80180-CRI-R2	AAACGTCGTTGGCCGCTTGTTGGC
MASS2-CRISPR	15400-CRI-F	GATTGAGCTTTCCGACATAGGCTT
	15400-CRI-R	AAACAAGCCTATGTCGGAAAGCTC
MASS3-CRISPR	20100-CRI-F	GATTGTGAAACGTAGCGCATCAGA
	20100-CRI-R	AAACTCTGATGCGCTACGTTTCAC
U6-sgRNA	pAtU6-F-EcoRI	ACGAATTCCATTCGGAGTTTTTGTATCTTGTTTC

	pAtU6-F-KpnI	GGGGTACCCATTCGGAGTTTTTGTATCTTGTTTC
	sgRNA-R-EcoRI	ACGAATTCGCCATTTGTCTGCAGAATTGGC
MASS1 promoter cloning	80180pro-F-EcoRI	CGGAATTCGTCTTTTCTCAGAACCAAAATATTCC
	80180pro-R-SacI	CCCGAGCTCGGACGGCGCTAACTGTTCCTCCTCCG
MASS2 promoter cloning	15400pro-F-EcoRI	CGGAATTCGACAATTGTATTTATGGATGGGCC
	15400pro-R-SacI	CCCGAGCTCGGATCTATCTCTCTCTCTCTGTTG
MASS3 promoter cloning	20100pro-F-NotI	ATAAGAATGCGGCCGCTTGAATTCGTCTCTAACTTTTAC
	20100pro-R-AscI	AGGCGCGCCCTTTTCTCTTCTCTCTTTCAATGGAATTG
MASS2 variant resistant to sgRNA targeting	M15400-R	CTTGACTCACTCATTGGTTTAGGTTGTTCGTCGCGTTG
	M15400-F	ACCAATGAGTGAGTCAAGTGAACAGGTCAAACCAATC
