# Supplementary material for: The MAPK substrate MASS proteins regulate stomatal development in Arabidopsis
Source: PLoS Genet. 2020 Apr 2;16(4):e1008706. doi: 10.1371/journal.pgen.1008706 (PMC7156110; doi:10.1371/journal.pgen.1008706)
Supplement: Attachment — Submitted filename: R1 respones_final.pdf [file pgen.1008706.s012.pdf]

There are a few major issues that need to be addressed in the next version of the manuscript.

Response:
We thank the constructive suggestions from the editor and the reviewers.

First, please discuss your findings in the context of previously published literatures (e.g. Sorensson et al., Biochem J., 2012).

Response:
We added more description and discussion about the previous publication (see nee additions in Lines 112-115, 129-131, 275-276 and 323-328)

Overall, Sorensson et al. focused a large-scale survey of MAPK substrates in Arabidopsis and by using MASS1 as an example to show this substrate could possibly regulate stomatal development. On the other hand, our work focused on detailed functional characterization of the MASS family (3 members) for their biological roles and the possible regulations with the MAPK signaling stomatal development.

Our research was initially inspired by the publication of Sorensson et al., Biochem J., 2012. Their major findings *related to our story* are **1)** it described the phosphorylation regulation of At1g80180 (referred as MASS1 in our research) by MAPKs; **2)** by brief overexpression assay, they suggested possible roles of MASS1 in the regulation of stomatal development. we believe that our current manuscript extended the research significantly in many dimensions: 1) established the phenotypes of the loss-of-function mutants; 2) established the overexpression plants consistently suggesting the positive role of MASS in stomatal production; 3) performed in-depth investigation of protein subcellular localization of the MASS proteins; 4) finely characterized domain structure of the MASS proteins and assigned their possible biological regulations; 5) connected protein phosphorylation status to MASS protein localization and function; and 6) identified the functional connection between MASS and the MAPKKK YDA.

The biological function of MASS1 in Sorensson et al. was not supported by in-depth functional genetic analyses, for example the loss-of-function mutants. In addition, some of their results were not fully consistent with our more detailed investigation that was briefly summarized above. The major improvements presented in our manuscript include:

1. Sorensson et al. did no characterize the loss-of-function mutants. We see this as a fundamental defect and essential for establishing gene function in modern plant molecular biology. In our manuscript, we created both T-DNA and CRISPR/Cas9 mutants and they gave

equivalent phenotypes. The genetic defects were also complemented by the *MASS* transgenes.

2. In Sorensson et al., we find the phenotypic characterization of the overexpression lines was NOT sufficiently thorough.
    a. As stated in the paper, they evaluated **2** individual T1 plants for phenotypic analysis. We did not find this number sufficient to make a solid conclusion. Our general practice is to characterize multiple independent T2 populations (at least 2 among 12 events) and at least 12 individual plants need to be examined for phenotypic quantification before we can establish the phenotype.
    b. Transgene expression levels should be evaluated and combined for phenotypic analysis in the overexpression studies. Sorensson et al. did not present RT-PCR or q-PCR to evaluate the phenotypes in their overexpression plants. In our manuscript, we completed all these assays.

Second, as YDA-MASS interaction is a key conclusion in this manuscript, we highly recommend that you perform Co-IP experiments to test their interaction in Arabidopsis.

Response:
We have unpublished data that the endogenous level of YDA-YFP expression in *yda* mutants was under detection by Western Blot. In addition, because the MAPKKK YDA is vital for plant development, overexpression of YDA suppresses stomatal formation and generates very weak and sick plants, which was a significant barrier for the suggested co-IP experiments in Arabidopsis.

Alternatively, we performed transient co-expression of YDA and MASS in tobacco plants and we now showed the successful co-IP data (see Figure 6D) supporting the YDA-MASS interaction in plant cells.

Additionally, we performed the Bio-Layer Interferometry (BLI) assay that is a label-free technology for measuring biomolecular interactions. In this experiment, we detected the physical interaction between recombinant proteins His-MASS2 and MBP-YDA (Figure. S7B).

Third, analysis of MASS proteins driven by their own promoters is highly recommended in some key experiments.

Response:
We have now added the localization pattern of the protein fusions driven by their own promoters that have complemented the loss-of-function mutants (MASS1pro::YFP-MASS1 and MASS2pro::GFP-MASS2, see below and Figure S3A and S3B)

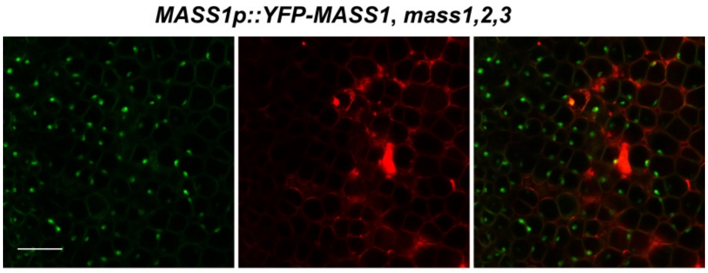

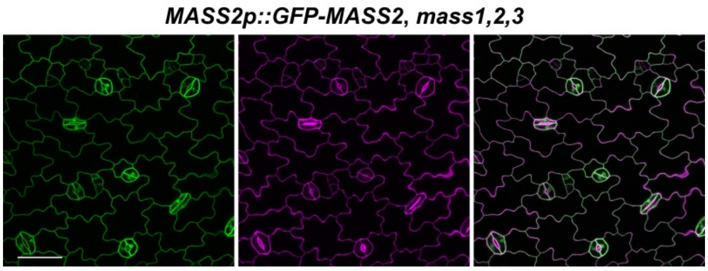

Fourth, some Z-projection data should be included in confocal analysis particularly in the cases where you exclude protein localization in some organelles.

Response: As suggested by the editor and reviewer, we have added the Z-projection data of YFP-MASS1 and YFP-MASS1-S105D into Figure S5A to provide more details about their differential subcellular localization (one example shown below).

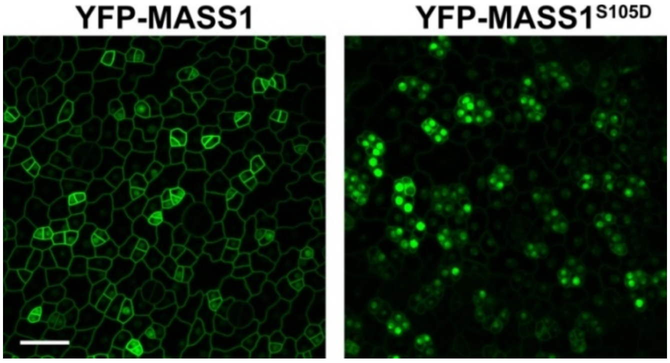

In addition, it is important to understand the effects of the YDA-MASS interaction pertaining to the questions like whether this interaction affects subcellular localization of YDA or MASS proteins, and how this interaction suppresses the inhibitory function of YDA in stomatal development.

Response:
With regards to the effect of defective YDA MAPK signaling, whether MASS localization is changed. We tried both strategies 1) overexpression of the

dominant negative *MPK6* and 2) chemically inducible inhibition of *MPK3*/6 in Arabidopsis, both of which generated super stomatal overproduction. The MASS1/2 subcellular localizations were not visibly changed (see the images below and new Figure S6A-S6C). It is possible that MPK3 and 6 signaling leakage was sufficient to phosphorylate MASS proteins and their PM localization. Alternatively, other kinases in the absence of *MPK3/6* may regulate MASS phosphorylation and function *in vivo*.

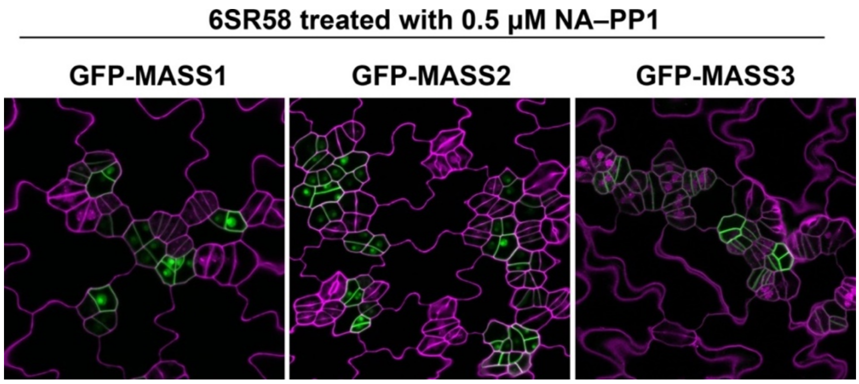

With regards to the subcellular localization and/or function of YDA, whether they could be modulated by the YDA-MASS interaction. The subcellular localization of YDA was not obviously changed by MASS2 overexpression (Figure 7A). In addition, we performed *in vitro* kinase assays, in which autophosporylation levels of YDA^{CA} were monitored in the presence of absence of MASS2. Our results (below and (Figure S7D) showed that no obvious changes were detected when MASS2 was in presence. There are many possibilities to interpret this: 1) The MASS-binding may change 3-D conformation of the YDA protein; 2) MASS may recruit other proteins to indirectly regulate YDA activity, function and interaction with other proteins. There are exciting research topics but outside of the scope of this work. Considering the mild stomatal phenotype generated by the triple loss-of-function mutations (Fig 2), the regulation of MASS on YDA is anticipated to be mild or elegant. Certain growth or environmental conditions might also help with better detection of the YDA MAPK signaling changes, which require dedicated effort for the exploration of another research direction.

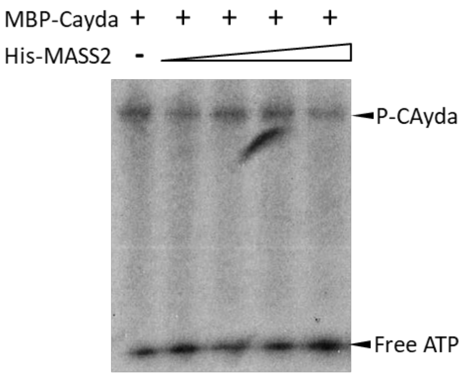

Reviewer #1:
Xue et al. reports a family of MAP Kinase substrate proteins, MASS proteins, that positively regulate stomatal development. The most striking finding is plasma membrane (PM) -nucleus localization dynamics of MASS and the demonstration that PM-localized MASS confers functions. All of these remind of the subcellular dynamics and MAPK regulation of BASL, which have been extensively studied by the authors' group, although the regulatory relationship between MASS proteins and BASL are not addressed in this manuscript.

The microscopy images presented are very beautiful, and the manuscript adds very interesting family of proteins to plant specific cellular polarity modules. Having said that, there are several critical issues that need to be addressed in order to improve the manuscript.

Response:
We thank the reviewer's positive comments and constructive suggestions. We made every effort to address the issues raised here.

1) Sorensson et al. report: First of all, the authors should properly acknowledge the previous work done by Sorensson et al. (Biochem J. 2012), who reported the identification of At1g80180 MASS protein (and its related protein At1g15400, now named MASS2) as in vitro and in planta MPK3/6 substrates that positively regulates stomatal development. Sorensson et al. have done quite in-depth experiments beyond this Xue et al. manuscript mentions. For example, Sorensson et al. have identified the MPK3/6 phosphosites of MASS1, performed site-directed mutagenesis to show site S105 is the phosphosite; the in vivo phosphorylation of MASS2 was reported by Scott Peck's group; 2004 Plant Cell, which makes Xue's phosphorylation experiments (Fig. 5A) rather confirmatory.

Response:
To further acknowledge the previous work by Sorensson et al., we added more description in the "Introduction" (Line 112-115), "Results" (Line 129-131, 275-276 and 323-328) and "Discussion" (Line 383-384) parts about their results. The reason that previously we were conserved about elaborating their results in our manuscript was that 1) our research was much focused on protein subcellular localization and the functional connection with the YDA MAPK signaling pathway, and 2) some of their conclusions were not fully consistently with our results, in particular regarding the phosphorylation consequence to MASS function (see more details below).

We did not find the evidence from the 2004 Plant Cell paper by the Peck group stating the phosphorylation of MASS2. The phosphoproteomic database http://plantsp.sdsc.edu is no longer accessible. Therefore, we could not cite this

paper. But we cited another study by the Waltraud Schulze group that suggested MASS2 being phosphorylated at the PM (Niittyla et. al., 2007).

More importantly, Sorensson et al. performed overexpression study of MASS protein, and reported that overexpressed phosphomimic version (S105D) confers stomatal clustering and increased stomatal index in Arabidopsis. This further diminishes Xie et al.'s Result section (line 122- 1st paragraph; Figure 1), making it largely confirmatory, and the authors' statement "we noted that one striking phenotype caused by MASS overexpression was clustered stomata" (lines 407-408) highly misleading, as it has been reported previously.

Response:
The discoveries reported in Sorensson et al. indeed initiated our interest (as a "stomata" research group) to further study the functions of the MASS family. But, our investigation as manifested in the manuscript has significant improvement in many dimensions, including the creation of loss-of-function mutants, characterization of protein subcellular localization, fine-scale analysis of protein subdomains, and functional connection with the YDA MAPK signaling pathway, etc. We believe these new discoveries give important insights about functions of the MASS gene family and will be greatly valuable to the field.

We also have two major concerns about some of the data reported by Sörensson et al. in particular the phenotypes created by overexpression of phosphomimic MASS1(S105D). First, their conclusion was based on the examination of two individual T1 transformants. According to our standard, this would not be sufficient for making any solid statement on a gene function. In practice, we need multiple T2 populations (12 independent lines) and sufficient number of individual plants (>12 from each established population) for phenotype quantification. Second, the overexpression protein fusion used in Sörensson et al. was MASS1(S105D)-HA. Based on our data, the C-terminal YFP fusion might interfere the C-terminal motif critical for protein function (see Figure 4).

As suggested, we replaced "striking" with "major" to avoid anything misleading.

Third, Sorensson et al. has reported the uniform promoter activity of MASS in developing cotyledons using MASSpro::GUS construct. The MASSpro::nucYFP expression analysis (Fig. S1) does not provide much more insight--- What new here is that Xue et al. also characterized the promoter activity of MASS2 and MASS3.

Response:
In general, we prefer to use GFP/YFP-tagged endogenous proteins to study expression pattern because these fusions provide more details about cell type-specificity. In this study, we not only generated the three *MASS* promoters

driving the expression of nuclear YFP, now also provided the expression and localization of the endogenous protein fusions (YFP/GFP/mCherry) of MASS1 and MASS2 that complemented the triple mutants (Figure2 and Figure S3A and S3B). Again, both genes were expressed in the leaves, but showed preferred cell type-specificity.

Thus, the major conclusion of MASS proteins as positive regulator of stomatal development and MPK3/6 substrates have been reported. Here, Xue et al. have done more careful analysis using sophisticated confocal microscopy and beautiful images—however, the manuscript needs to be re-written and re-organized to properly and fairly acknowledge the previous findings

Response: We thank the constructive suggestions made by the reviewer. We have added more descriptions to further acknowledge the previous findings by Sorensson et al. (Line 112-115, 129-131, 275-276 and 323-328). We also highlighted the differences of our findings compared with the previous publication (Line 323-328.)

> "Compared to the previous discoveries by Sorensson et al. that phosphomimick MASS1 trigged more stomatal production, our findings suggested that phosphorylation of MASS2 is critical for protein localization and, more interestingly, the reversible phosphorylation-dephosphorylation might be more important for MASS2 function."

2) Lack of analysis using endogenously expressed MASS proteins: Here all experiments of subcellular localization and interaction studies are done using ectopic promoters in the stomatal cell lineage promoters (e.g. 35S promoter, BASL promoter, SPCH promoter—Data presented in Figs. 1,3, 4,5,6,7). While use of ectopic promoters will provide "cleaner" results to interpret data-thus could be a powerful tool, however, caution should be made as it is after all ectopic expression.

Response: We have now added the endogenous protein localization of MASS1 and MASS2 (new Fig. S3A and S3B), the expression of which complemented the loss-of-function mutants.

MASS1p::YFP-MASS1, mass1,2,3

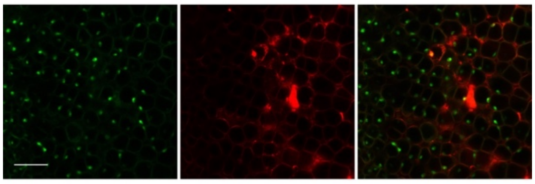

MASS2p::GFP-MASS2, mass1,2,3

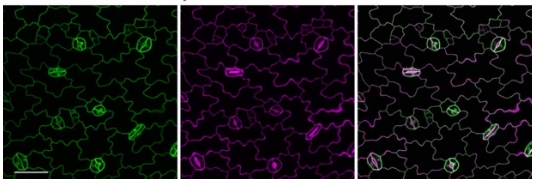

I don't see any YFP signal in Fig. 2C when the endogenous promoter was used (MASS1/2/3pro::YFP-MASS1/2/3). Is the endogenous expression that low?

Response: Fig. 2C was not intended to show protein localization, but to demonstrate that the N terminal-tagged MASS1/2 proteins complemented the triple mutants. Now, the protein expression for MASS1/2 were added in new Figure S3A and S3B.

Do the BASLpro:MASS1/2/3 complement the modest low stomatal index phenotype of mass1/2/3? Or does it still constitutively-active and causes excessive stomatal lineage divisions? If the latter, I would be very cautious in interpreting the subcellular localization.

Response:
We did not introduce BASLpro:MASS1/2/3 into the triple mutants. Only endogenous promoter-protein fusions were used for complementation. All the transgene expression using the BASL promoter were assayed for protein subcellular localization and overexpression phenotype in stomatal development.

Based on Fig. S1B, the promoter activity of MASS1-3 are much higher in pavement cells (and they are not expressed in stomatal precursor cells and stomatal guard cells). Where do MASS proteins localize in the pavement cells?

Response:
We now added the subcellular localization patter of MASS1 and MASS2 (Fig. S3A and S3B). Although MASS1 showed most abundant expression in the mesophyll cells, both proteins maintained comparable localization patterns in pavement cells in tobacco (Fig 3A-3C) and Arabidopsis (Fig. S3A and S3B). We also added a sentence in the main text to clarify this point (Line 187-190).

3) YDA-MASS interactions: The novelty (and exciting findings) of this manuscript lies in subcellular localization dynamics and precise mapping of MASS in YDA-MAPK cascade. The YDA-MASS association are tested in in vitro pull down, BiFC and Y2H (Fig. 6A-C), all highly sensitive yet occasionally face false positives, and lacking in vivo subcellular contexts. The co-localiation of YDA and MASS in plasma membrane speckle is interesting, however, again, ectopic promoters (SPCH promoter and BASL promoter) are used for expressing MASS and YDA. Overexpression of fluorescent tagged proteins are known to often form aggregates, and I think it is important for the authors to perform Co-IP experiments of YDA-MASS interactions using endogenously expressed (i.e. epitope-tagged proteins driven by the endogenous promoter) YDA and MASS.

Response:
We appreciate the reviewer's constructive suggestions.

We have unpublished data that the endogenous level of YDA-YFP expression in *yda* mutants was under detection by Western Blot. In addition, because the MAPKKK YDA is vital for plant development, overexpression of YDA suppresses stomatal formation and generates very weak and sick plants, which was a significant barrier for the suggested co-IP experiments in Arabidopsis.

Alternatively, we performed transient co-expression of YDA and MASS in tobacco plants and we now showed the successful co-IP data (see below and Figure 6D) supporting the YDA-MASS interaction in plant cells.

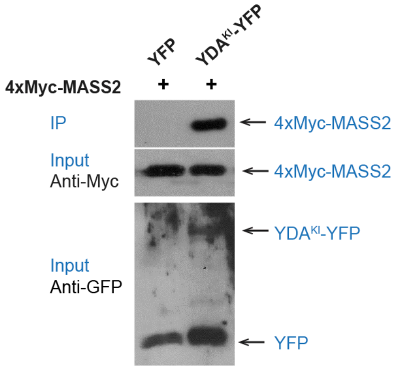

Additionally, we performed the Bio-Layer Interferometry (BLI) assay that is a label-free technology for measuring biomolecular interactions. In this experiment, we detected the physical interaction between recombinant proteins His-MASS2 and MBP-YDA (see below and Fig. S7B).

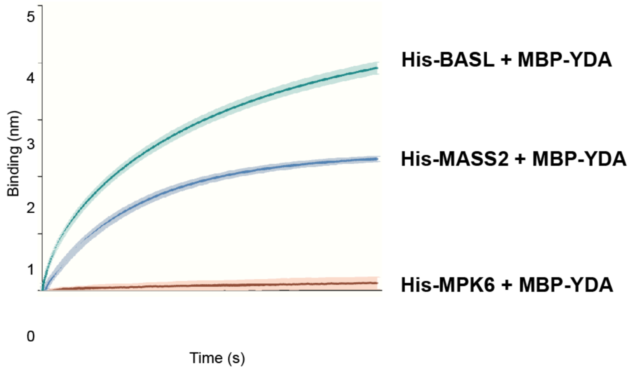

3) Genetic interactions of MASS with YDA-MAPK cascade: Again, the genetic studies are done with ectopic expression of MASS vs ectopic expression of YDA (Fig. 7), which could be misleading. The authors need to present genetic analysis using loss-of-function mutants, which properly places the components in the genetic pathway. Because the phenotype of mass1/2/3 triple loss-of-function mutants is so subtle/weak, it may be masked by strong yda loss-of-function phenotype. However, the authors could test mass1/2/3 interactions with mpk3 and mpk6. Since mpk3 and mpk6 single mutants have no stomatal phenotype (or slightly elevated stomatal index, as reported by Putarjunan et al. 2019), the mass1/2/3 could counteract with the modest elevated stomatal index phenotype of mpk3 and mpk6.

Response:
We agreed with the reviewer that the strong stomatal proliferation phenotype created by the loss-of-function *yda* or the double mutation of *mpk3*;6 would mask the mildly reduced stomatal density in *mass1;2;3* triple mutants. Thus, creation of higher order mutants between *mass* and *yda, mpk3*;6 may not be informative.

On the other hand, combining *mass1;2;3* with *mpk3* or *mpk6* single mutants would also involve other complications, such as functional compensation between MPK3 and 6 observed in *Arabidopsis*. Wang et al. (2008 Plant Cell) reported the haplo-insufficiency of *MPK3* in *mpk6* mutants in ovule development and detected elevated activity levels of MPK6 in *mpk3* mutants. In addition, the MAPK cascade is well known for signal transduction to trigger cellular responds in response to external cues. This means that developmental phenotypes of the single mutants may vary under different growth conditions. In addition, practically speaking, establishment of the quadruple mutants of *mass1;2;3;mpk3* and *mass1;2;3;mpk6* will take significant time and effort that may not necessarily lead to clear delineation of the genetic interactions because of the complications discussed above. Taken together, we are not convinced that the creation of such higher order mutants would give significant improvements of the study.

4) Subcellular localization of MASS proteins in YDA-MAPK mutants: The authors mention that neigher phosphomimic or phosphonull substitutions of MASS proteins properly localize to PM (Fig. 5; lines 266-268). What are the subcellular localizations of YFP-MASS1/2/3 in mpk3/6 double and YDA mutants? This need to be characterized to confirm that YDA-MPK3/6 are indeed required for proper PM localization of MASS.

Response:
We have tried multiple assays attempting to detect possible localization change of MASS1 and MASS2g when MPK3 and 6 signaling were disturbed. We found that MASS1/2/3 still maintained their PM localization when MAPK signaling is disturbed by overexpression of the dominant negative (kinase inactive) MPK6 or by chemically inducible inhibition of MPK6 in *mpk3;6* null mutants (Fig. S6A-S6C and representative data shown below). It is possible that these genetic backgrounds still have MPK3 and 6 signaling leakage, so that MASS maintains phosphorylation and PM localization. The other possibility is that other kinases other than MAPKs may simultaneously phosphorylate MASS *in vivo*.

Also, we have added comments about these possibilities in the Results part (see lines 300-302 and the new Fig S6C as shown below).

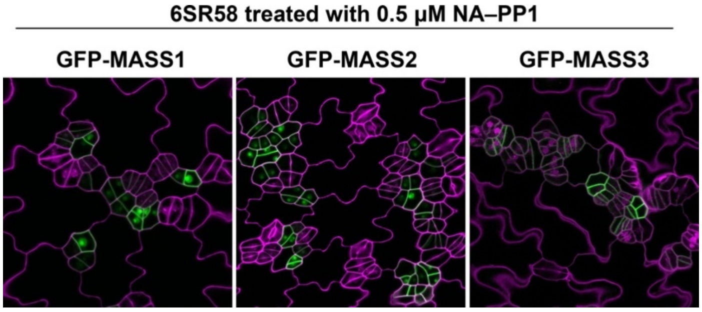

5) Positive role of MAPK: In the Intro (and at some extent in Discussion), the authors rationalize their study mentioning that MAPK pathway components that positively regulate stomatal development is not well studied (lines 77-79). The Lampard et al. 2009 and 2014 Plant Cell papers show that the YDA-MKK7/9-MPK3/6 module promotes stomatal development when expressed in the later stomatal precursor cells (GMC-to young guard cells, driven by the FAMA promoter). None of the expression studies of Xue et al. manuscript are done in the later stage of stomatal precursors. As far as I can see from Fig. S1, the MASS1/2/3 promoters are not even active in the GMC-young GCs. How could the authors imply the role of MASS proteins in the known positive pathway?

Response:
We appreciate the reviewer's deep understanding of the MAPK signaling in the regulation of stomatal development. As the exact mechanism for the YDA-MKK7/9-MPK3/6 pathway to promote stomatal production is unknown,

MASS proteins are MPK3/6 substrates and MASS2 was indeed expressed in mature stomatal stage (see Figure S3B), we discussed the possible functional connection in the previous version. Considering that this speculation was not felt sufficiently justified by the reviewer and possibly the audience, we have deleted this part in the Discussion to avoid anything misleading.

6) Intro-ICE1: Recent study identifies ICE1 as a scaffold to recruit MPK3/6 to nucleus, to bridge to SPCH, which does not physically associate with MPK3/6 (Putarjunan et al., 2019). Thus, the Introduction mentioning that “ICE1 is also phosphorylated by MPK3/6 (line 86-)” (and also related sentence in Discussion) is not properly reflecting the current knowledge.

Response:
We thank the reviewer for critical reading of the manuscript. The citation has been added into the Introduction (Lines 88-89)

Minor: Fig. 5A “CCB” should be “CBB”?

Response:
We thank the reviewer for careful reading of the manuscript. This has now been corrected.

Reviewer #2:
The authors present a considerable body of work examining the role of MASS proteins in stomatal development. Along with mutant and overexpressors, the study examines localization and motif analysis as well as protein-interaction studies. The study concludes that the MASS proteins represent novel positive regulators of stomatal development that act in a localization dependent manner to inhibit the activity of the MAPKKK YDA. Overall, this body of work presents novel insight into the MAPK module that regulates stomatal development and is clearly present and discussed. There are however, components of the study that require clarification or expansion to fully justify the author's conclusions.

Minor comments
SI graphs would be better presented as violin plots or box and whiskers to show the distribution of the data. qRT-PCR expression analysis graphs should also show the data points. Both of these are relatively common requests with regards presentation.

Response:
We thank the reviewer for constructive suggestions. The histograms have been converted to blox plots. qPCR data now contain fold changes in the figures.

P8 Line 145 '….mutant produced lowered density of stomatal guard cells.' Here the authors are referring to SI data and so this should reflect that this is a proportion and not density.

Response:
Agreed. This is now rephrased to ".. mutant produced lowered stomatal index".

Figure 2C/F. The authors refer to the complementation lines in the insertion mutant but the SI data for these lines is missing from 2F.

Response:
The quantification for complementation assay were conducted on the *mass1;2;3-crispr#7* plants and supported by two independent lines (see Fig. 2G). The complementation of T-DNA triple mutants was shown with images (Figure 2C) but not quantified.

The various fusion proteins and the resulting microscopy is given as proof of localization. For clarity, it would be good to see some blots probed with a GFP-antibody to demonstrate that these are fusions and that there is not significant free-GFP contributing to the signal.

Response:

We were not quite certain about the reviewer's suggestion. How would Western Blot further support the specific protein localization patterns? Typical GFP-only in the stomatal lineage cells shows diffused signals in the cytoplasm and nucleus and because enlarged pavement cells are highly vacuolated, the cytoplasmic signals on single optical sections by Confocal may appear close to the cell periphery (see image below, TMMpro::GFP). We are familiar with Cofocal imaging and it is easy to determine a specific protein subcellular localization or the non-specific signals. Also, the protein fusions were tested for their function in the loss-of-function mutants.

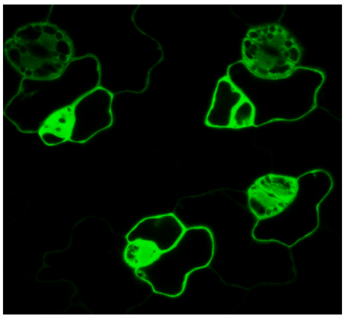

P15 line 295. Can you really not show the data here – if it resembles the CA-YDA then it would be good to see.

Response: We have now provided the whole-plant growth status of the triple mutant to compare with *yda*^{CA} plants (Figure S6D).

Major comments

Confocal imaging is vital here for demonstrating the localization but there is little detail in the methods regarding the image capture and post-capture processing. To demonstrate fully the different localisations seen, some Z-projections are required in supplemental data. This is particular the case when the argument is that the protein is only at the membrane or the nucleus – a single plane is not sufficient to demonstrate this.

Response:

We have now provided the comparison between single optical section images and z-projection images (see Figure S5). All the confocal images we provided for the figures in the manuscript are representative of hundreds to thousands of cells that we have observed.

Interactions with YDA. Personally, for in planta interaction, a Co-Ip would be stronger proof than the in vitro method (not in planta), BiFC or the co-localisation. Certainly, these two latter results could potentially be explained by interactions with MPK6 and this being in close proximity to YDA.

Response:
We have unpublished data that the endogenous level of YDA-YFP expression in *yda* mutants was under detection by Western Blot. In addition, because the MAPKKK YDA is vital for plant development, overexpression of YDA suppresses stomatal formation and generates very weak and sick plants, which was a significant barrier for the suggested co-IP experiments in Arabidopsis.

Alternatively, we performed transient co-expression of YDA and MASS in tobacco plants and we now showed the successful co-IP data (see new Figure 6D) supporting the YDA-MASS interaction in plant cells.

Additionally, we performed the Bio-Layer Interferometry (BLI) assay that is a label-free technology for measuring biomolecular interactions. In this experiment, we detected the physical interaction between recombinant proteins His-MASS2 and MBP-YDA (see new Fig. S7B).

The authors utilize the correct methodology to examine the role of phosphorylation (generating phosphomimics and mutants). However, the results are confusing. I'd like to add that the authors have been clear in reporting this and it's good to see these results reported. However, I think that the resulting interpretation is only one possible explanation. If the argument is that phosphorylation is important for targeting then yes, you would hope to see differential localization. The alternative explanation is that the amino acid changes have both altered function independent of any role of phosphorylation.
What would perhaps be useful in resolving this would be an analysis of the CFP-MASS2 localisation when crossed with the constitutively active version of YDA (7a). Here, you would at least expect enhanced MPK6 activity and MASS phosphorylation – so do you see an overall change in membrane versus nuclear localization?

Response:
We thank the reviewer for sharing the informative insights.

Yes. It is indeed possible that the amino acid changes altered MASS function independent of the role of phosphorylation. We have now added this new comment in the Results part (see Lines 302-306).

Align with the reviewer's suggestion, it would be helpful to have *in vivo* evidence to show how modulation of the YDA MAPK signaling may change the subcellular localization of MASS, though we were more in favor of testing the negative impact. As a matter of fact, we have tried multiple assays attempting to detect possible localization change of MASS1 and MASS2g when the YDA downstream MPK3 and 6 signaling are disturbed. We found that MASS1/2/3 still maintained their PM

localization when MAPK signaling is disturbed by overexpression of the dominant negative (kinase inactive) MPK6 or by chemically inducible inhibition of MPK6 in *mpk3;6* null mutants (Fig. S6 and representative data shown below). It is possible that these genetic backgrounds still have MPK3 and 6 signaling leakage, so that MASS maintains phosphorylation and PM localization. The other possibility is that other kinases other than MAPKs may simultaneously phosphorylate MASS *in vivo*.

Also, we have added new comments about these possibilities in the Results part (see lines 300-302 and new Fig. S6C).

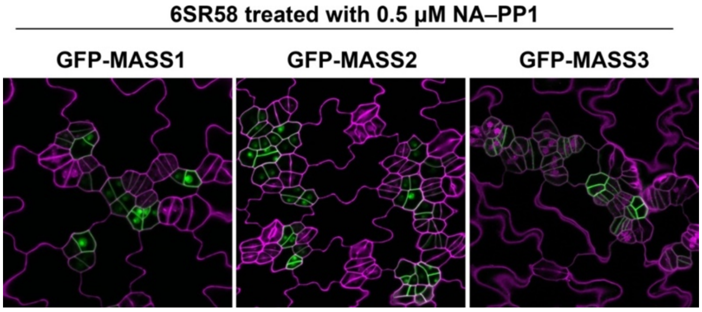

On a similar front. If the expectation is that these MASS proteins inhibit YDA and therefore activation of the MAPK pathway, you might expect to see higher basal MPK6 activity in the mass triple mutant and lower MPK6 activity in the OE lines – this can be tested with phosphor specific antibodies. This could also be correlated with gene expression analysis of SPCH and some of its targets.

Response:
We now have provided new data that MPK3/6 activities are elevated in the triple mutants and suppressed in the overexpression MASS2 plants (see below and new Fig. S7C)

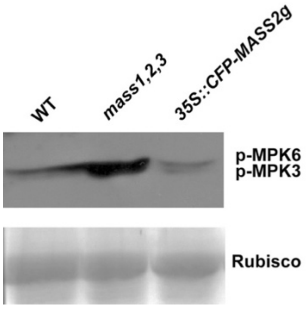

In their discussion, the differential localization of BASL is discussed and the similarities drawn with the localization of the MASS proteins. Linking to above, a question that arises is therefore, where is MPK6 phosophorylation these MASS proteins? In the nucleus or is it the cytoplasm or when they associate with the PM? The author's discussion suggest they favour a nuclear phosphorylation event. I'm not suggesting that they determine this though I believe that they have the tools to

do so. However, they could therefore slightly expand their discussion here to discuss the relevance to signalling of this model – they suggest it is a pool for responding to signals but the implications are that this would be a potential feedback mechanism to bring the MAPK signal back to normal. The question would then be what the stability is of the MASS proteins at the PM and whether they are dephosphorylated and then re-targetted or just broken down. Basically, their model could be expanded on.

Response:
We appreciated the reviewer’s constructive suggestion.

We now added new comments on the possibility of MASS being phosphorylated in the cytoplasm and at the PM (Line 457-461).

The reviewer brought up an excellent point about the stability of the MASS protein at the PM. We now have added both new discussion and expanded the working model as shown in (Figure 7C).

Reviewer #3:
In their manuscript, Xue et al., identified a novel family of small proteins that are involved in stomatal development. A member of this 3-protein family named MASS was previously found to be a substrate of MPK3/6. Through overexpression and mutant analyses, the authors first demonstrated that the MASS proteins act to promote stomatal production. Fluorescent reporters showed that the MASS proteins are localized either to both the plasma membrane and the nucleus or exclusively to the plasma membrane, and that they exert their function on the plasma membrane. Deletion and amino acid substitution analyses further identified that specific protein domains and the MPK6-targeted phosphorylation site on MASS2 are important for its subcellular localization and function. Finally, the authors found that MASS proteins physically interact with the triple MAP kinase YDA and appear to suppress the inhibitory function of YDA in stomatal development.

The YDA-mediated MAP kinase pathway is a key module in regulating stomatal development and patterning, as well as in other developmental processes. Thus, it is of great interest to understand how YDA is regulated. The identification of the MASS proteins as new stomatal regulators acting through YDA provides novel insights into the regulation of this critical signalling pathway. The functional dissection of the domains and the phosphorylation site also yielded valuable information about these proteins. In addition, the quality of data and the writing are generally good. To further strengthen manuscript, here are a few points that I would like the authors to address:

Response:
We thank the reviewer's positive comments. We made every effort to address all the issues raised here.

1. The authors mentioned that the overexpression of MASS proteins produced "overproliferated early stomatal lineage… cells" (Line 131) but did not show any quantitative data. Since these "early lineage cells" are highly relevant to YDA activity, I think the authors should quantify them in young cotyledons/leaves in the overexpression and the triple mutants.

Response:
We thank the reviewer for the constructive advice. Since the quantification data we presented in the manuscript mainly included stomatal index and clustered stomatal index. To expand the quantification to "early stomatal lineage cells", we will have to redo all the numbers for 4 main figures, which requires significant effort and time. We decided to rephrase this line in the main text to "all the transgenic populations produced similar stomatal phenotypes: overproliferated stomatal guard cells in a clustered pattern" (Line 137-139).

2. The stomatal index data for mass1;2;3-crispr#5 and rescued lines were mentioned but are missing (Line 164 and Line 172).

Response:
We have amended the quantification of stomatal index of *mass1;2;3-crispr#5* (new Fig. S2D). The complementation assay, as mentioned in the main text, was performed in the *mass1;2;3-crispr#7* background, therefore we quantified two independent line of MASS2p::mCherry-rMASS2g in *mass1;2;3-crispr#7* in the main figure (Fig. 2G*)*.

3. For the BiFC (Fig. 6C and S5A), there should be another negative control with nYFP and YDA-KI-CFP.

Response:
As suggested, we have amended another negative control, nYFP + YDA^{KI}-cYFP (Fig. 6C).

4. The kinase inactive YDA was previously shown to cause a dominant negative phenotype (i.e. over-proliferation of the stomatal lineage) (Lampard et al., 2009). If the authors have the relevant data, I think the authors could indicate whether the overexpression of MASS2g (Fig. 6D) can further enhance the dominant negative phenotype.

Response:
We have the images. But it is difficult to make a conclusion that overexpression of MASS2g (Fig. 7A) can further enhance the YDA dominant negative phenotype. Because the stomatal clustering phenotype in the YDA dominant negative background was so strong that masks possible additive phenotypes, such as the one generated by MASS2 overexpression.

5. Since the authors are the first to characterize this protein family, it would be helpful to the community to indicate if the mutants have other obvious growth defects and if these proteins are expressed in other tissues or developmental stage.

Response:
We thank the reviewer for the constructive suggestion. Now we have included figures to show discernable promoter activities of the three *MASS* genes in other plant tissues (Fig. S1B). Indeed, they are broadly expressed at the seedling stage

despite different levels were found. A statement was also added into the main text (Lines 146-148).

In the *mass* triple mutant population, we occasionally found cotyledon fusion at the young seedling stage (Fig. S6D), suggesting their additional functions in leaf development.

Others:
- The AGI code for MASS3 in the text (Line128) and the figure is not correct (compared with Line 452)

Response:
Thanks for careful reading of the manuscript. We apologize for the negligence. These have now been corrected.

- In Fig. 6B, the authors should indicate more clearly in the figure or legend that MBP and MBP-YDA were used as baits.

Response:
Thanks for careful reading of the manuscript. We have added more description in the figure and legend.
